# Supplementary material for: Tailored antisense oligonucleotides for ultrarare CNS diseases: An experience-based best practice framework for individual patient evaluation
Source: Mol Ther Nucleic Acids. 2025 Jul 1;36(3):102615. doi: 10.1016/j.omtn.2025.102615 (PMC12302494; doi:10.1016/j.omtn.2025.102615)
Supplement: Document S1. Tailored antisense oligonucleotides for ultrarare CNS diseases [file mmc1.pdf]

OMTN, Volume *36*

Supplemental information

Tailored antisense oligonucleotides for ultrarare CNS diseases: An experience-based best practice framework for individual patient evaluation

Rebecca Schüle, Holm Graessner, Annemieke Aartsma-Rus, Willeke M.C. van Roon-Mom, N=1 Collaborative (N1C), 1 Mutation 1 Medicine Consortium (1M1M), and Matthis Synofzik

Supplemental Material

1M1M Screening dossier.

The screening dossier is designed to enable a first rapid screening evaluation whether a submitted case is in principle eligible for an individualized ASO therapy approach, considering properties of the variant, disease, and patient. After positive evaluation of the screening dossier, a more detailed case evaluation is performed based on the full dossier.

1M1M Full dossier.

The full dossier is designed to capture all key information that is required to perform an individual benefit-risk evaluation, determining the suitability of the respective individual for an individualized treatment approach. The depth of information goes considerably beyond the information provided in the screening dossier. Drawing on the information provided here, a multi-stakeholder gene group will deliberate on whether the current data is adequate for the evaluation process by a treatment board. If deemed insufficient, the group will identify the specific types of additional information required.
