# Supplementary material for: Tailored antisense oligonucleotides for ultrarare CNS diseases: An experience-based best practice framework for individual patient evaluation
Source: Mol Ther Nucleic Acids. 2025 Jul 1;36(3):102615. doi: 10.1016/j.omtn.2025.102615 (PMC12302494; doi:10.1016/j.omtn.2025.102615)
Supplement: Document S2. 1M1M screening dossier [file mmc2.pdf]

1M1M Screening Dossier

The screening dossier is designed to enable a first rapid screening evaluation whether a submitted case is *in principle* eligible for an individualized RNA therapy approach, considering properties of the variant, disease, and patient. After positive evaluation of the screening dossier, a more detailed case evaluation is performed based on the full dossier.

Please give information for all topics in the following tables.

**Variant information**	
Gene	
Genomic variant position (GRCh38)	
cDNA variant(s)<br>*In case of compound-heterozygosity, please underline the ASO target variant*	
Functional variant effect<br>*Please describe the variant effect on RNA and protein level*	
Reference(s)<br>*If the variant has been published, please provide a reference.*	

**Disease information**	
Please describe the main phenotype(s) associated with variants in this gene. Which neuronal system/organ is typically most severely affected?<br>*Provide references to key publications, OMIM etc.*	
Briefly describe the typical course of the disease.<br>*Please comment on age of onset, major disability milestones (provide approximate age), life expectancy.*	-

**Patient information**	
Please describe the current functional status of your patient.<br>*Please briefly comment on the functional status of each key functionally relevant health aspect, e.g. cognitive functions, ability to communicate, mobility, upper limb function, independence in activities of daily living etc.*<br>*Which neuronal systems/organs are mainly affected in your patient?*<br>*If data on your patient has been published, please provide a reference.*	

Please complete all fields of the screening dossier and send it to: 1M1M@med.uni-tuebingen.de

1M1M Full Dossier

This full dossier is designed to capture all key information that is required to perform an individual benefit-risk evaluation, determining the suitability of the respective individual for an individualized treatment approach. The depth of information goes considerably beyond the information provided in the screening dossier. Drawing on the information provided here, a multi-stakeholder gene group will deliberate on whether the current data is adequate for the evaluation process by a treatment board. If deemed insufficient, the group will identify the specific types of additional information required.

**Variant and ASO strategy information**	
In the following, please provide additional information on the variant you propose to target with an ASO.	
Gene	
Genomic variant position (GRCh38)	
cDNA variant (→ ASO target)<br>*Provide the reference transcript.*	
Variant frequency (GnomAD v4)	
Functional variant effect<br>*Please describe the variant effect on RNA and protein level.*	
Proposed ASO strategy<br>*e.g. downregulation, splice-modulation, etc.*	
Status of the preclinical development	
Reference(s)<br>*Please provide references to relevant publications, e.g. on the variant, functional studies on the relevant mutational mechanism, ASO development for this or other variants in the gene etc.*	

**Disease information**	
Please add general information on the target disease (if available: mutation-specific). Give references if possible.	
Age of onset spectrum	
Please describe the main phenotype(s) associated with mutations in this gene. Which organ is typically most severely affected?	
Please describe the natural course of the disease. If no formal natural history is available, please describe disease milestones (e.g. loss of walking ability, loss of ability to communicate, blindness, …)	

Prognosis regarding <u>morbidity and mortality</u>	
Which <u>therapeutic options</u> are currently available for this disease? Please list approved treatments as well treatments currently under development	

**Patient information**	
Please use this paragraph to give current information on the patient(s) carrying the variant described above. If there is more than one patient carrying the same potentially treatable variant, please copy and paste the patient information table to accommodate information about additional patient(s).	
Identifier	
Clinical contact person	
Last examination / update of clinical information (date)	
Genotype	
Family history	
Month/year of birth	
Age of onset	
Weight	
Hight	
Sex	
Patient location (city, country)	
Patient history (brief description)	
Summary of the phenotype	
Co-morbidities	
<u>Current</u> functional stage	
Affected organs (liver, kidney, CNS, PNS, muscle, skeletal, integumentary, cardiovascular, pulmonary, other)	

Primary goal of therapy	
Which relevant medications have been tried?	
Current medication	
Availability of patient-derived cell lines (fibroblasts, lymphozytes)	
Aspects that might interfere with compliance	

**Outcomes**	
Please propose outcomes that may be used for measuring the ASO treatment effect. Which outcomes (clinician-reported outcomes, patient-reported outcomes, performance-related outcomes, digital-motor (body-worn sensors), imaging (MRI) and molecular outcomes (e.g. neurofilament light chain)) are available that might be used in a clinical trial/for a treatment? The proposed outcomes should be prioritized according to their likelihood to capture change on a single subject level. The proposed set of outcomes should include outcomes which clearly reflect - either directly (e.g. PROMs) or indirectly (e.g. biomarkers) - patient-meaningful aspects of health.	
Clinician-Reported outcomes (ClinROs)	
Performance Related Outcomes	
Patient-reported outcomes	
Biomarker outcomes (molecular, imaging, etc)	
Please also provide information on the question: which functions will the patient likely loose in the next 3-5 years?	

**Available Resources**	
What resources are available for the preclinical and clinical development of the ASO treatment? Resources may include ongoing projects, funding sources, existing collaborations, …	
Preclinical development	
ASO manufacturing	
Clinical development	

Potential additional patients carrying the target variant
Please use this section to suggest, how additional patients carrying the target variant might be identified (e.g. contact to diagnostic centers, collaborators, authors of relevant publications, databases etc.)

Please complete all fields of the dossier and send it to: 1M1M@med.uni-tuebingen.de
