# Supplementary material for: Tailored antisense oligonucleotides for ultrarare CNS diseases: An experience-based best practice framework for individual patient evaluation
Source: Mol Ther Nucleic Acids. 2025 Jul 1;36(3):102615. doi: 10.1016/j.omtn.2025.102615 (PMC12302494; doi:10.1016/j.omtn.2025.102615)
Supplement: Document S3. Article plus supplemental information [file mmc3.pdf]

Molecular Therapy
Nucleic Acids
Clinical Perspective

Tailored antisense oligonucleotides for ultrarare CNS diseases: An experience-based best practice framework for individual patient evaluation

Rebecca Schüle,[1,2] Holm Graessner,[3,4] Annemieke Aartsma-Rus,[5] Willeke M.C. van Roon-Mom,[5] N=1 Collaborative (N1C), 1 Mutation 1 Medicine Consortium (1M1M), and Matthis Synofzik[6,7]

[1]Division of Neurodegenerative Diseases and Movement Disorders, Department of Neurology, Heidelberg University Hospital and Faculty of Medicine, Heidelberg, Germany; [2]Center for Neurology and Hertie Institute for Clinical Brain Research, University of Tübingen, Tübingen, Germany; [3]Institute of Medical Genetics and Applied Genomics, University of Tübingen, Tübingen, Germany; [4]Center for Rare Diseases, University Hospital Tübingen, Tübingen, Germany; [5]Department of Human Genetics, Leiden University Medical Center, Leiden, the Netherlands; [6]German Center for Neurodegenerative Diseases (DZNE), Tübingen, Germany; [7]Division Translational Genomics of Neurodegenerative Diseases, Hertie-Institute for Clinical Brain Research and Center of Neurology, University of Tübingen, Tübingen, Germany

Individualized mutation-specific RNA therapies offer promise, in particular, for individuals with ultrarare neurological diseases that affect only few families or even single patients worldwide. Outside traditional drug development pathways, however, clinicians and scientists face the challenge of systematically evaluating whether individual patients with severe ultrarare diseases might be eligible for and potentially benefit from such approaches. This complex evaluation involves biological, clinical, psychological, and ethical aspects. Based on the experience of the 1 Mutation 1 Medicine (1M1M) consortium, we here propose a best practice framework that enables the systematic evaluation of individual patients for tailored genomic therapies, using transparent criteria to comprehensively assess each individual's benefit-risk balance. By example of individually tailored antisense oligonucleotide approaches in neurology, this framework takes into account characteristics of the (1) underlying variant, (2) underlying disease, and (3) individual patient. It thereby allows a systematic, balanced, fact-based evaluation that appreciates the full complexity and preferences of each individual patient, performed by a multi-stakeholder treatment board. This operational framework will thus pave the way for systematic, rational patient-centric evaluation and decision-making in the rapidly evolving field of individualized "n-of-1" precision genomic medicine in clinical neurology.

INTRODUCTION

Breakthrough DNA and RNA therapeutic approaches using nucleic acid editing, modulating, and replacement techniques successfully transitioned from bench to bedside, enabling effective therapies for previously untreatable diseases as heterogeneous as B cell lymphoma (lisocabtagene maraleucel),[1–3] metachromatic leukodystrophy (atidarsagene autotemcel),[4–6] amyloidosis (inotersen, patisiran),[7–13] amyotrophic lateral sclerosis (tofersen),[14–16] or spinal muscular atrophy (nusinersen, onasemnogene abeparvovec).[17–21] Many of these therapeutic technologies are based on platform approaches for genetic modification which keep key modules of drug development consistent, while other parts can be customized to a specific disease or variant. Offering significant acceleration and efficiencies, these platform technologies are thus inherently characterized by the potential to treat (even rare and ultrarare) disease mutations and genes at scale.

Genetic neurological diseases are often progressive in nature, cause considerable morbidity and mortality, and mostly lack specific therapeutic options. As such, they are in principle ideal candidates for these novel therapeutic approaches. However, the majority of genetic neurological diseases or mutations are ultrarare with a prevalence several orders of magnitude below the threshold defining rare diseases (prevalence for rare diseases: <1:2,000 [European Union]; prevalence for ultrarare diseases: <1:1,000,000 [Orphanet][22]).

Traditional drug development pathways are suitable for more common diseases proceeding through phase 1 through 3 trials toward marketing authorization. Orphan regulatory frameworks—created by administrations around the globe to incentivize development of therapies for rare diseases—have facilitated drug development for the more common rare diseases. Nevertheless, they have had limited success in advancing therapies for ultrarare diseases due to multiple reasons, including lack of established methodology to demonstrate

https://doi.org/10.1016/j.omtn.2025.102615.

Correspondence: Rebecca Schüle, Division of Neurodegenerative Diseases and Movement Disorders, Department of Neurology, Heidelberg University Hospital and Faculty of Medicine, 69120 Heidelberg, Germany.
E-mail: rebecca.schuele@uni-heidelberg.de
Correspondence: Matthis Synofzik, Research Division Translational Genomics of Neurodegenerative Diseases, Hertie-Institute for Clinical Brain Research and Center of Neurology, University of Tübingen, Hoppe-Seyler-Str. 3, 72076 Tübingen, Germany.
E-mail: matthis.synofzik@uni-tuebingen.de

© 2025 The Author(s). Published by Elsevier Inc. on behalf of The American Society of Gene and Cell Therapy.
This is an open access article under the CC BY license (http://creativecommons.org/licenses/by/4.0/).

efficacy, lack of appropriate regulatory frameworks, and limited commercial opportunity, altogether providing an increased risk for drug developers[23] without obvious straightforward reward.

Regulators define "severely debilitating or life-threatening" diseases or conditions (SDLTs) as those which cause major irreversible morbidity and/or in which the risk of death is high (21CFR312.81). For these diseases, urgent drug development programs are required, with much faster bench-to-bedside development timelines unachievable by traditional drug development routes. Regulators are starting to formulate "out of the regulatory box" access routes for SDLTs that are ultrarare (often called "n-of-1" or "n-of-few" cases), e.g., the named patient program and first specific statements from the European Medicines Agency (EMA) in form of scientific protocol advice for Antisense Oligonucleotide (ASO) treatment programs[24] in Europe, the investigational new drug program in the United States,[25] and a series of draft guidances on n-of-1 drug development for SDLTs by the Food and Drug Administration (FDA).[26–29]

ASOs are single stranded, chemically modified, synthetically produced RNA molecules that can be designed to bind to virtually any endogenous RNA sequence by Watson-Crick base pairing. ASOs are a highly promising therapeutic modality for ultrarare indications due to their programmable nature; relatively easy and cost-effective chemical synthesis; comparably good tissue penetrance especially in the liver and, after local injection, in the central nervous system (CNS) and eye; and often favorable benefit-risk profile (for recent reviews on ASO mode of action, chemistries, and development aspects see previous studies[30–34]). For CNS application, maintenance dosing regimens involve intrathecal injections every 3–4 months. Depending on various chemical modifications, ASOs can either induce degradation of their RNA target (gapmer, e.g., inotersen[8] or tofersen[15]), block access of splicing factors to the bound sequence, and thus modify RNA splicing (steric hindrance mechanism, e.g., nusinersen[19]) or block protein translation (e.g., VO659 [NCT05822908]). Effectiveness of intrathecal ASO therapies in neurological diseases has been demonstrated, e.g., by nusinersen, an approved splice-modulating ASO that acts to increase production of the survival motor neuron (SMN) protein using RNA steric hindrance during splicing, overall resulting in a dramatic slowing of clinical disease progression of spinal muscular atrophy.[19]

As proof of principle for advancing the ASO technology of nusinersen to individualized application, the academically driven development of Milasen, an intrathecally delivered ASO targeting a unique, deep-intronic cryptic splice site in a girl with Batten's disease (*CLN7*),[35] has demonstrated the applicability of this platform therapeutic approach to a single case. It has stimulated formation of global multi-stakeholder initiatives promoting advancement of so-called n-of-1 disease-modifying genetic therapies, including the "1 Mutation 1 Medicine" consortium (1M1M; https://www.1mutation1medicine.eu/), the N = 1 Collaborative (N1C; https://www.n1collaborative.org/), and the n-Lorem Foundation (https://www.nlorem.org).

Embarking on such n-of-1 and n-of-few drug development pathways outside traditional drug development is, however, challenging and resource intensive and carries specific clinical, economic, and ethical risks compared to many other investigational programs. Determining whether an individual patient qualifies, the likelihood of benefit, as well as accounting for individual heterogeneity in disease presentation and diversity of preferences, poses significant challenges. This evaluation involves a complex mélange of biological, clinical, psychological, and ethical aspects. Drawing on our collective experience in evaluating numerous patients for individualized ASO therapies, the 1M1M consortium proposes a best practice framework for assessing patient eligibility for tailored ASO treatments. This framework is based on transparent criteria that support a comprehensive evaluation of the individual benefit-risk balance. The 1M1M framework takes into account characteristics of the (1) underlying genetic variant, (2) underlying disease, as well as (3) individual patient. Although developed for intrathecal ASO therapy of CNS diseases, many aspects of the framework can be extrapolated to other diseases and treatment modalities within the rapidly evolving field of individualized genomic medicine and its scalable platform approaches (see Table 1). Due to its operational nature and multi-stakeholder involvement in the evaluation and decision processes, the framework will moreover serve to alleviate pressure and reduce bias from individual clinicians, scientists, patients, and their families. It thus promotes an objective, balanced, fact-oriented approach taking into account the full complexity of each individual case. We are fully aware that we are learning as our experience with these novel treatments grows. The patient identification framework is therefore dynamic, reflecting the current state-of-the art developed by working groups of the 1M1M and N1C consortia and will continue to be adapted and improved to incorporate new information, knowledge, and experience.

THE 1M1M FRAMEWORK FOR INDIVIDUAL PATIENT EVALUATION: OVERVIEW

To evaluate whether patients might have a sufficiently high likelihood to experience a net benefit from individualized, experimental ASO therapies, individual patients are assessed within the framework by criteria on three axes (Figures 1A and 1B).

(1) Is the patient's disease-causing variant suitable for the proposed therapeutic ASO strategy? (*variant & ASO strategy criteria*)
(2) Is the patient's disease well suited for a tailored RNA treatment approach? (*disease criteria*);
(3) Are the patient's individual characteristics well suited for this therapy approach? (*patient criteria*)

Each axis is evaluated according to inclusion and exclusion criteria, which support the identification of a potential candidate variant, disease, and patient, respectively. If all criteria are evaluated favorably, additional weighting factors may influence the individual benefit-risk ratio. Based on the assessment of each of the three axes, an overall summary evaluation of the benefit-risk balance of the therapeutic approach in the respective patient can be drawn. This is performed

Table 1. Potential application domains of the 1M1M framework in genomic medicine: Targeted treatment modalities for ultrarare diseases and mutations

Therapeutic modality	Development status
**1. RNA therapies (targeting or delivering RNA)**	
a) Oligonucleotide therapies (non-coding; chemicals)	
Splice modulation ASOs	first tailored mutation/exon-specific splice modulation ASOs in clinical application, e.g., CLN7 (Milasen),[35] CLN3 (Zebronkysen),[36] or Ataxia Teleangiectasia (Atipeksen)[37]; many additional in advanced preclinical development by academic investigators and industry, e.g., by 1M1M and n-Lorem[38]
Allele-specific or biallelic gapmer ASOs	first tailored gapmer ASOs in clinical application, e.g., SCNA2 (Elsunersen),[39] KIF1A,[40] TNPO2 (Leosen)[41]; or KCNT1 (Valeriasen[41]), many in advanced preclinical development by academic investigators and industry, e.g., by n-Lorem[38]
RNA editing	preclinical development for CNS disorders such as amytrophic lateral sclerosis (A-to-I RNA editing, via ADAR2-Cas13 editing of GluA2[42]), Rett syndrome, and other epileptic encephalopathies (ADAR recruitment [industry]). Plus phase 1/2 trial (ACDN01) for retinal disorder ABCA4 (Stargardt disease.[43] RNA exon editor delivered via AAV)
siRNA	in phase 1 clinical trials for rare neurological diseases such as Huntington's disease (ALN-HTT02 [NCT06585449])[44] and spinocerebellar ataxia type (NCT06672445) and other rare CNS diseases. High potential also for ultrarare CNS diseases and mutations.<br>first siRNA drug approved by FDA (2018) for hereditary transthyretin-mediated amyloidosis with polyneuropathy (hATTR), a rare genetic disorder (Patisiran 2018, later also Vutririsan 2022)
b) Coding RNA (biologicals)	
mRNA	under development for rare neurogenetic diseases, (1) with systemic metabolic defects that can be addressed by targeting the liver or (2) that require direct targeting of nervous tissues (for review, see Monfrini et al.[45])<br>in clinical trials for methylmalonic acidemia (MMA) (mRNA-3704 [NCT04899310]), propionic acidemia (PA) (NCT04159103); in preclinical development for phenylketonuria (PKU), arginase 1 (ARG1) deficiency, Gaucher disease,[46] and Friedreich's ataxia (FRDA[45]).
**2. Gene therapies (targeting DNA)**	
Gene replacement (e.g., via viral vectors)	in clinical development for ultrarare diseases, e.g., SPG50[47] or Tay Sachs disease[48]; development also of platform approaches, e.g., platform vector gene therapies (PaVe-GT) program[49,50]
in vivo gene editing (e.g., via CRISPR-Cas9	first in clinical use for patient-specific mutations (e.g., CPS1 deficiency[51]). Plus in preclinical testing (rodent models) for several more common neurogenetic diseases, e.g., ALS[52] or Huntington's disease[53]

Many of these technologies rely on platform approaches for genetic modification, in which key components of the drug development process remain consistent, while other elements can be tailored to a specific disease or genetic variant. This modularity enables scalable treatment of ultrarare mutations and genes. For an overview of splice modulation or gapmer ASOs and their respective development status, see Chen et al.[54] (additional rare inherited metabolic diseases) and Stern et al.[55] (more common rare diseases that have been approved or are in first-in-human trials).

by a multi-stakeholder treatment board, which establishes an independent evaluative perspective on the overall benefits and risks. However, the treatment decision needs to be ultimately based on the patient's (or their caregivers') evaluation.

VARIANT AND ASO STRATEGY CRITERIA

First and foremost, causality of a candidate variant for the phenotype needs to be established unequivocally (e.g., American College of Medical Genetics and Genomics [ACMG] class 4 or higher[56]) and suitability of the variant for a promising ASO therapeutic approach needs to be established, hereby ensuring that the selected strategy appropriately addresses the underlying mutational mechanism. This constitutes the main inclusion criterion on the variant and ASO strategy axis (Figure 2).

The flexible design of ASOs in terms of sequence and chemistry, including modifications of the phosphate backbone nucleobase or sugar ring, allow for their precise "programming" to target a wide range of variants and mutational mechanisms.[58] *Gapmer ASO designs* rely on activation of cytoplasmic RNase H enzymes that degrade the ASO-RNA heteroduplex and result in downregulation of the protein translated from the targeted transcript. Gapmers are therefore typically applied to target transcripts containing gain-of-function mutations, variants acting via a dominant-negative mode of action or key proteins in a pathological pathway. In contrast, ASOs acting via *steric hindrance* include modifications to the sugar ring that inhibit RNase activation. They can be targeted to the translation initiation codon or splice regulatory motifs to inhibit protein translation or modulate splicing. ASO-mediated splice modulation hereby is a particularly powerful and flexible tool and can be used in a wide range of applications including correction of cryptic exonic or intronic gain-of-splice variants, exon skipping or inclusion approaches and "targeted augmentation of nuclear gene output" (TANGO), a method licensed by Stoke Therapeutics to increase protein translation from non-productive transcripts for potential use in diseases of haploinsufficiency.[57]

Depending on the selected interventional ASO strategy, additional conditions may need to be met. *Cryptic splice variants*—variants located in the exon or intron that lead to activation of cryptic splice sites and thus aberrant splicing—are excellent targets for splice-modulating ASOs as their correction can lead to expression of fully

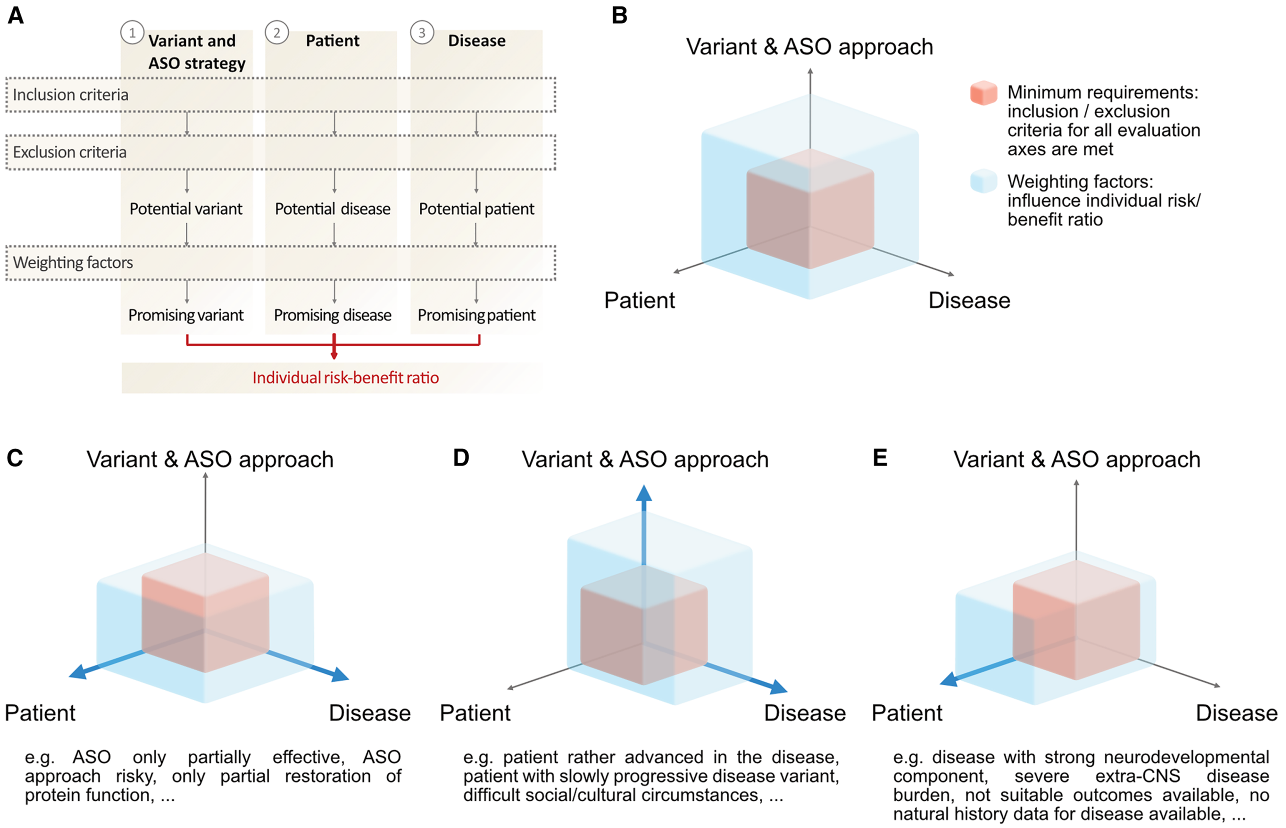

Figure 1. Evaluation strategy

(A) Each case scenario is evaluated along three criteria axes by systematically applying inclusion and exclusion criteria as well as additional weighting factors. Eventually, an overall summary evaluation of the individual benefit-risk ratio is reached. (B–E) Graphic illustration of the evaluation process (B) with three hypothetical case scenarios (C–E). The benefit-risk evaluation along each of the three criteria axes varies from case to case (blue shape) but should never fall short of the minimum requirements visualized by the red shape. The weighting axis is emphasized by a blue line. CNS, central nervous system.

functional, wild-type protein. However, if they are located too close to the exon-intron boundary (rule of thumb: <15 bp), ASO-mediated inactivation of the cryptic splice site may be impossible without simultaneous impact on the neighboring canonical splice site, thus resulting in skipping of a constitutively spliced exon. Steric hindrance ASOs can also be targeted to splice enhancer or suppressor motifs and thus modulate splicing in versatile ways. Notably, current strategies are unable to correct effects of canonical splice site variants.

Exon skipping of constitutively spliced exons is often more limited. If the mutation-containing exon is out of frame, skipping this exon would lead to a shift in the open reading frame and thus result in nonsense mediated decay of the transcript or translation of a dysfunctional protein. Even for mutations of in-frame exons, applicability may be limited by the functional necessity of specific protein domains encoded by the targeted exon. Successful implementation of an exon skipping strategy therefore requires prior demonstration of not only at least partial restoration of protein expression and key disease-relevant protein functions by skipping of the mutant exon but also *in vivo* evidence—from model systems or the human disease—that this partial restoration is likely to result in a milder phenotype.[59,60]

When using ASOs to *downregulate expression of transcripts or proteins* containing gain-of-function or dominant-negative variants either by translational arrest or induction of RNase degradation of the targeted transcript, insufficient remaining wild-type protein expression (haploinsufficiency) can be a problem. If it is demonstrated that complete loss of the target protein leads to a milder (or absent) disease phenotype compared to expression of the mutant protein, haploinsufficiency likely is not a concern. However, when reduction of protein levels is associated with a phenotype, allele-specific ASOs targeting only the mutant transcripts have to be considered.

In addition to these basic considerations that determine the fundamental feasibility of a mutation-specific ASO therapy for a specific gene defect, several supplemental factors, referenced in Figure 2 as "weighting factors," may influence the benefit-risk balance. Prior knowledge about the expected mechanistic efficiency of the ASO

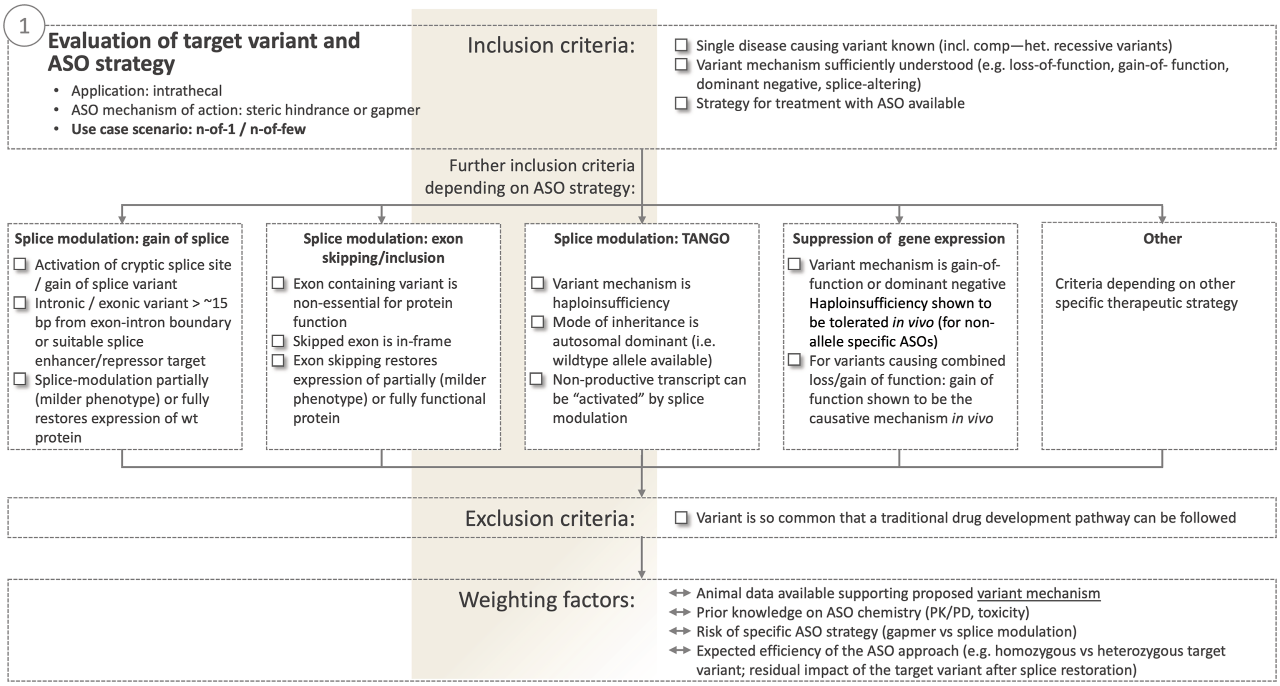

Figure 2. Evaluation of the target variant and ASO strategy

Undisputed causality of the targeted variant for the phenotype and an ASO strategy that addresses the underlying mutational mechanism are key inclusion criteria on the variant axis. Depending on the selected ASO strategy, further inclusion criteria may need to be considered. comp.-het., compound-heterozygous; PK/PD, pharmacokinetic/pharmacodynamic; WT, wild-type; TANGO, targeted augmentation of nuclear gene output.[57]

might be available, e.g., when targeting a homozygous rather than a heterozygous ASO-treatable variant or if preliminary preclinical evidence is already available. On the risk side, prior knowledge about chemistry-related toxicity, e.g., when using a well-studied backbone and modifications, or choosing a splice-modulating approach over a gapmer approach due to the often more favorable clinical safety profile of splice-modulating ASOs might be considered risk-mitigating.

All of these considerations are *per se* independent of the frequency of the genetic variant targeted by an ASO approach. However, when intending to develop an ASO for a single or very few cases with an SDLT, rapid development of the therapy is paramount due to the severe and progressive nature of the disease (see below). Therefore—unless already available—prospectively obtaining sufficient functional evidence in *in vivo* models to support, e.g., exon skipping or expression reduction approaches might often be too time consuming. This again underlines the special position of cryptic splice variants in this context: since here expression of the wildtype protein can be restored via inactivation of the cryptic splice site, ASO therapies of cryptic splice sites can usually be implemented more rapidly on an n-of-1 level than other ASO-mediated therapeutic strategies.

In summary, evaluation of variants for ASO treatment require careful weighting of detailed knowledge about the mutational mechanism and ASO modes of action in the context of the disease biology. The process therefore typically relies on the combined expertise of geneticists, clinicians, and experts on the disease protein as well as ASO therapies.

DISEASE CRITERIA

To maximize the chances of benefiting from the intrathecal application of a customized ASO, it is crucial that the disease pathophysiology primarily affects the brain or spinal cord (including anterior horn lower motoneurons), rather than peripheral nerves or non-neurological systems such as the eyes, liver, or hematological systems (DI2; for overview on all criteria on the disease level, see Figure 3). Sufficient evidence is required to substantiate that the brain/spinal cord regions driving the respective CNS disease are indeed reached by intrathecal delivery of the ASO (Figure 3: criterion DI4). This highlights the urgent need to acquire and publish more comprehensive human postmortem data on brain and spinal cord distribution of therapeutic ASOs, e.g., the gapmers used in the recent trials in Huntington's disease (GENERATION HD1 Trial, NCT03761849; Tabrizi et al.[61]) and SOD1 ALS (VALOR trial, NCT02623699; Miller et al.[15]). Yet, initial postmortem human brain pathology data show a widespread brain and spinal cord distribution for splice-modulating ASOs with a Nusinersen backbone—albeit with a clear gradient from lumbar via thoracic spine to brainstem and motor cortex.[19] Rodent and non-human primate data, moreover, suggest a similarly widespread distribution for RNase H ASOs, including, e.g., basal ganglia and deep cerebellar nuclei.[62]

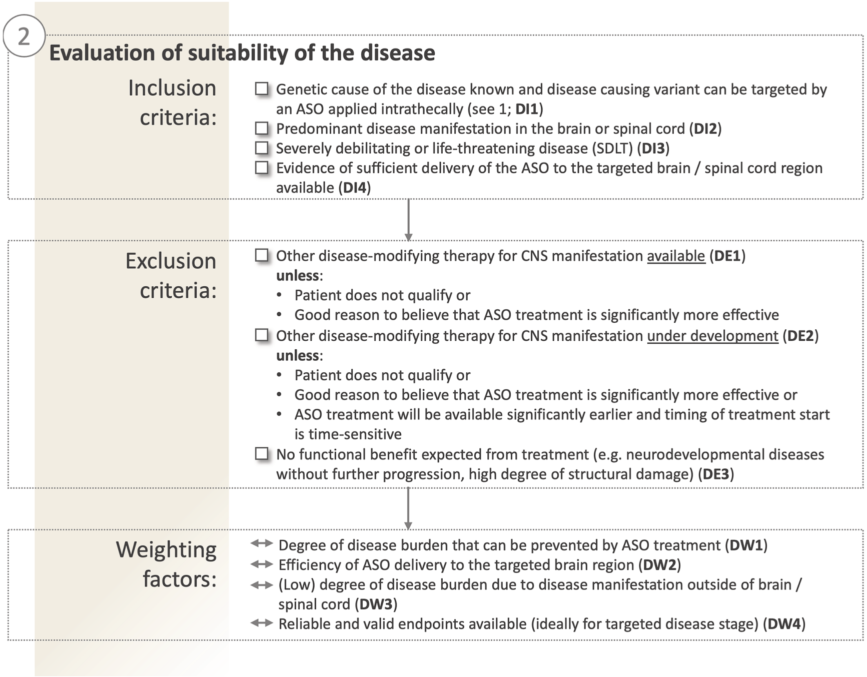

Figure 3. Evaluation of the suitability of the disease
The severely debilitating and life-threatening nature of the disease, main disease manifestation in the CNS, and absence of other effective therapeutic options are key inclusion criteria on the disease axis. Disease inclusion (DI) and exclusion (DE) criteria as well as disease-related weighting criteria (DW) are numbered in the figure and referenced throughout the text. CNS, central nervous system; SDLT, severely debilitating or life-threatening disease.

To justify an expedited, non-classical drug development route and regulatory setting, only diseases that meet the definition of a "severely debilitating or life-threatening disease or condition" (SDLT) can currently be considered (DI3) in order to balance the inherent risks of an experimental drug development and application with the risk of no treatment. Universally accepted, objective and specific criteria to define SDLTs (21CFR312.81) are missing. A recent review by Liu et al., however, may provide some pragmatic guidance and suggests the following criteria to define "irreversible morbidity."[63]

(1) Major disability leading to significant reduction in health-related quality of life and activities of daily living (e.g., permanent loss of independence in performing daily activities, poor neurological health interfering with activities of daily living);
(2) Progressive worsening of a condition leading to potential for only incompletely reversible or irreversible significant functional impairment, morbidity or death, without adequate therapies or other options to control disease activity.
(3) Recurrent hospitalizations resulting from a severely debilitating condition (e.g., greater than one hospitalization annually) for life-threatening events (i.e., major organ system dysfunction or failure, significant injury).

A net benefit from the individualized CNS ASO therapy for such SDLTs is particularly likely if no other effective disease-modifying therapy are available. If an alternative therapy option is already available, a net benefit from the individualized CNS ASO therapy might still result if the newly developed ASO is likely to yield a high add-on effect to the existing therapy, is likely to be considerably more effective, or associated with significantly lower risks (DE1 and DE2).

Careful evaluation of disease evolution and pathomechanisms is also required to evaluate whether substantial functional benefit can still in principle be expected upon ASO treatment (DE3). In particular for neurodevelopmental diseases without further relevant progression, the neuronal damage might already have happened at the time ASO treatment is considered (usually postnatally or in early infancy), starting an ASO therapy would be unlikely to restore the existing damage. Yet, here the benefit of improved future development trajectories enabled by ASO therapy might still be considered in the context of remaining neuronal plasticity which might allow functional benefit.

ILLUSTRATION OF DISEASE CRITERIA

The aforementioned disease considerations in principle can serve as binary inclusion/exclusion criteria to assess the fundamental eligibility of a given disease. However, in practice, we frequently encounter gray zones when the criteria mentioned above are only partially met. The criteria then function as weighting factors that influence assessment of the likely benefit-risk ratio of the candidate disease (Figure 1B). In this context, a relatively high degree of disease burden that can potentially be prevented by ASO treatment (DW1), an efficient delivery of the ASO to the targeted brain region (DW2), and a low degree of disease burden outside of brain/spinal cord (DW3) can support the suitability of an SDLT for a tailored treatment approach. Additionally, availability of reliable and valid efficacy endpoints, ideally with sensitivity to (treatment-induced) change even on a single-subject level, can be considered a supportive weighting factor (DW4). In particular, both efficacy and safety biomarkers can provide important data in deciding early in the treatment process whether or not treatment should continue, particularly since clinical outcome measures might be more noisy or slow to change.

The following examples, all real-world scenarios evaluated by 1M1M in recent months, demonstrate practical application of the proposed disease criteria. (1) Mutations in *PLP1*, some of which may meet variant axis criteria, give rise to a variety of allelic phenotypes: for instance, Pelizaeus-Merzbacher disease is associated with a high

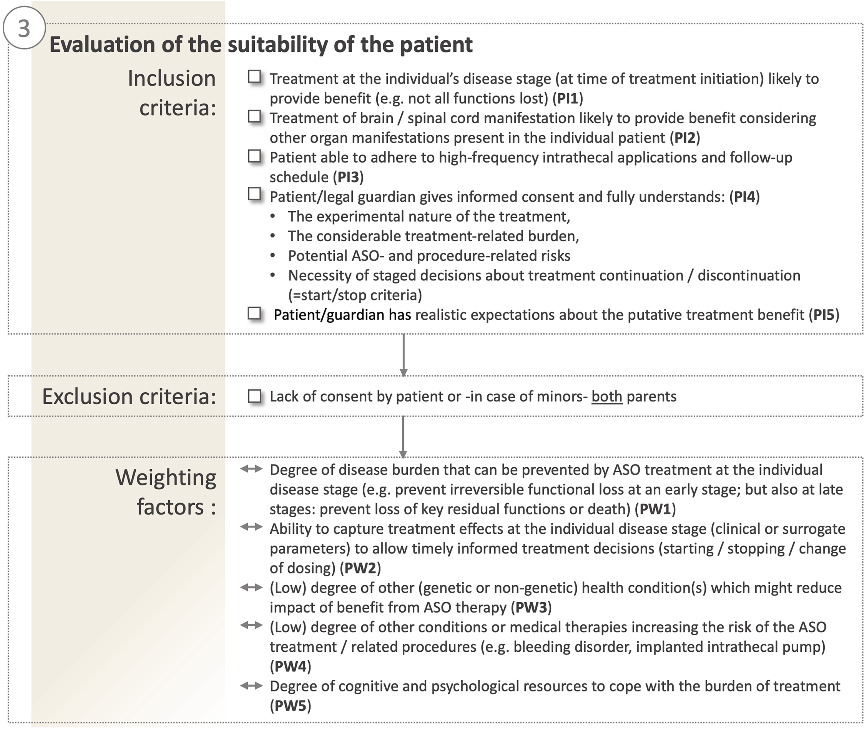

Figure 4. Evaluation of the suitability of the patient
Evaluation of patient-related criteria involves rational fact-based criteria as well as strong consideration of individual patient preferences. Patient inclusion (PI) and exclusion (PE) criteria as well as patient-related weithing criteria (PW) are numbered in the figure and referenced throughout the text.

disease burden and reduced lifespan. Meanwhile, hypomyelination of early myelinating structures (HEMS) on the other hand presents with a considerably milder phenotype and progression and thus may not qualify as SDLT[64] (DI3). Moreover, the slow progression of HEMS also complicates the choice of endpoints with sufficient sensitivity to change within reasonable time frames to allow decisions on the efficacy of an individualized ASO treatment protocol (DW4). Thus, even within a given disease gene, the specific allelic phenotype may result in divergent suitability for individualized ASO development. (2) Wiedemann-Rautenstrauch syndrome (WRS) is a severe neonatal progeroid disease syndrome with intrauterine growth retardation, failure to thrive, short stature, and variable mental impairment. WRS leads to extreme functional impairment, complete dependence and average survival of 7 months, clearly establishing it as an SDLT[65] (DI3). It can be caused by specific deep-intronic cryptic splice variants in *POLR3A*[66] (variant criteria met). However, despite the progressive, fatal disease course, most of the neuronal (and extra-neuronal) damage occurs neurodevelopmentally *in utero*. Postnatal ASO therapy is highly unlikely to restore this damage (DE3). In contrast, other deep-intronic mutations in *POLR3A* give rise to a later-onset predominantly neurodegenerative spastic ataxia phenotype,[67] which might be more suited for an ASO therapeutic approach. (3) Ataxia telangiectasia (AT) is a mostly degenerative condition leading to progressive cerebellar ataxia and other movement disorders. In up to 15% of AT patients at least one cryptic splice mutation eligible for a splice-modulating ASO approach can be identified,[37] thus passing the variant criteria. Classic AT usually leads to loss of ambulation in teenage years, and further loss of independence at 15–25 years of age, thus also meeting the SDLT criteria (DI3). Although AT includes substantial non-neurological systems damage (e.g., immunodeficiency, cancer), the main disease burden in terms of activities of daily living is driven by the CNS damage, thus also meeting this disease criterion of intrathecal ASO application (DI2). However, while functional disease burden is mainly driven by CNS damage, death mostly results from non-CNS causes, in particular cancer. As this non-CNS disease feature is not expected to be treated by the intrathecal ASO, this reduces the overall net benefit to be expected from the ASO approach. While this does not present an exclusion criterion, it exemplifies how the degree of expected net benefit is modulated by the described weighting factors, here: the relative degree of morbidity and mortality driven by non-CNS dysfunction in a respective candidate disease (DW1).

PATIENT CRITERIA

In the evaluation for personalized ASO development, the characteristics of the presenting patient—and not necessarily of the "average patient," like for conventional clinical trials—need to be uniquely factored into the eligibility evaluation (as well as into the treatment outcome design). Timing of treatment initiation relative to the individual disease stage and expected disease trajectory is critical (PI1; for overview of all criteria on the patient level, see Figure 4). Previous preclinical and clinical trials suggest that treatment effects might diminish or even completely vanish in advanced stages of neurodegenerative disease.[15,68] Recent trial designs for disease-modifying treatments accommodate this experience by focusing exclusively on early and/or mild disease stages, e.g., the A4 study evaluating amyloid-lowering treatments in elderly individuals at risk for memory loss (NCT02008357),[69] the AHEAD 3-45 study targeting preclinical AD (NCT04468659),[70] or a trial testing tofersen, an intrathecally applied ASO, for pre-symptomatic *SOD1* mutation carriers at risk for developing ALS (NCT04856982).[71] Very likely the same applies to individuals with an ultrarare mutation or disease. Treatment is thus ideally initiated as early as possible, but necessarily at a disease stage when potential structural damage and functional loss are not yet too advanced (PI1). Notably, additional challenges regarding timing of treatment, evaluation of efficacy and weighing of the individual risk-benefit-ratio arise when

considering treatment of pre-symptomatic individuals under an n-of-1 paradigm.

Secondly, multisystem involvement beyond the CNS is common in genetic ultrarare diseases. Intrathecal ASO application is unlikely to significantly influence disease manifestations outside of the CNS. Functionally predominant CNS—rather than extra-CNS—manifestations thus present an important criterion not only on a disease level (DI2), but also on an individual level (PI2). Not only may the individual disease manifestation vary across the phenotypic disease spectrum, but also the patient's preferences and conception of what is considered meaningful benefit may influence the evaluation.

Other inclusion criteria largely pertain to procedural aspects and the experimental nature of the therapy. Patients (and families) need to understand the treatment burden (repeated intrathecal injections; safety and efficacy monitoring), potential risks related to the specific treatment as well as the procedure, and the uncertain treatment benefit (PI3-5). As regulators are beginning to draft guidelines for n-of-1 safety and toxicity studies,[72–75] patients and investigators must be fully educated that the reduced preclinical package required before first-in-human dosing in the n-of-1 setting, although streamlined as part of a platform approach, may result in a less certainty on both safety and compared to traditionally developed drugs. Patients and their families therefore need to understand and accept this higher level of uncertainty. These issues may be difficult to understand in their full complexity for patients/guardians and may require a multi-stage information and consenting process.

ILLUSTRATION OF THE PATIENT CRITERIA

In real-life, the threshold when an individual is too severely impaired to justify an experimental ASO therapy can be hard to define. In very severely affected cases and advanced disease stages, the decision that treatment will likely not lead to any meaningful functional benefit can often be made with relative clarity. The criterion of individual disease severity then applies absolutely, as an exclusion criterion (PI1). This is exemplified by a woman with an advanced neurodegenerative multisystemic "optic atrophy plus" syndrome due to biallelic *OPA1* mutations, one of them a deep-intronic cryptic splice variant amenable to treatment with a splice-switching ASO (individual II.3 from family OAK587 described in Bonifert[76]). At age 50–48 years after disease onset, she had been wheelchair bound for 40 years already, was severely ataxic (Scale for the Assessment and Rating of Ataxia [SARA] score 34/40) and nearly blind. She was therefore determined not to be eligible for treatment as benefit was considered unlikely. Borderline cases are much more difficult to classify, and the disease stage criterion then acts as a weighing factor that might tip the final decision to one side or the other when evaluated in synopsis with variant and disease level criteria (PW1).

Similarly, the criterion of predominant CNS disease manifestation—prerequisite of efficacy of an intrathecally applied ASO—can act in a dual role as inclusion criterion as well as a weighting factor (PI2). For example, starting an intrathecal ASO treatment in a patient with AT and a manifest hematological malignancy poorly responding to treatment and severely limiting the life expectancy is likely futile (i.e., extra-CNS disease manifestation as an exclusion criterion). Yet another patient with AT, although still at heightened risk for developing a malignancy, might qualify for an ASO treatment if quality of life is mainly determined by the neurological disease burden which might be prevented by an ASO therapy (i.e., extra-CNS disease manifestation as a weighting criterion).

Moreover, caution is required when extrapolating general knowledge about the course of a disease to an individual patient. Many neurodegenerative diseases can manifest with a wide spectrum of phenotypes and progression trajectories and typically only part of this variability can be explained by the underlying disease-causing mutation.

SUMMARY EVALUATION

Following careful evaluation of the inclusion and exclusion criteria and potential weighting factors on each axis, an overall summary evaluation of the individual benefit-risk ratio of the proposed treatment is needed. The relative degree of likely benefit should here be integrated across all three axes (Figures 1A and 1B), and then be weighed against the treatment-related risks and burden to the patient and caregivers. The latter encompass not only risks associated with ASO treatment itself, like potential toxicity, or the taxing nature of administration, such as the necessity of lifelong lumbar punctures several times a year. They also involve additional challenges such as recurring demanding assessments to evaluate effectiveness and safety outcomes. Moreover, there's the requirement for repeated sedation during ASO administration and magnetic resonance imaging (MRI) procedures procedures for certain individuals and the need for recurrent hospitalizations. Such treatments also often imply a substantial psychosocial burden for the family, who need to reorganize their daily life settings around the required treatment protocol. Scarcity of clinical sites qualified to administer these treatments may even require relocation of the family to the treatment site.

The final evaluation of the equipoise between benefit and harm is ultimately not a descriptive, medical question; rather, it is normative in nature and determined by personal values of what is considered meaningful benefit and acceptable harm/risk of harm. Importantly, these norms and values do not need to be *ethical* norms, i.e., they do not need to have any claim of general validity; they can be *evaluative* norms based on one's personal conception of a "good."[77]

The role of the treatment board hereby is to derive a benefit-risk evaluation of the respective ASO treatment in a respective patient that is fact-based and experience-driven, but will always also include evaluative norms. Thus, to avoid potentially implicit normative bias driven by personal or professional evaluative norms, this evaluation should be developed by a multi-stakeholder team considering multiple individuals and perspectives such as treating clinicians, non-treating (i.e., not primarily involved, potentially more "neutral") clinicians, other professionals and patient representatives. Based on this evaluation, the treatment board will decide whether to offer a

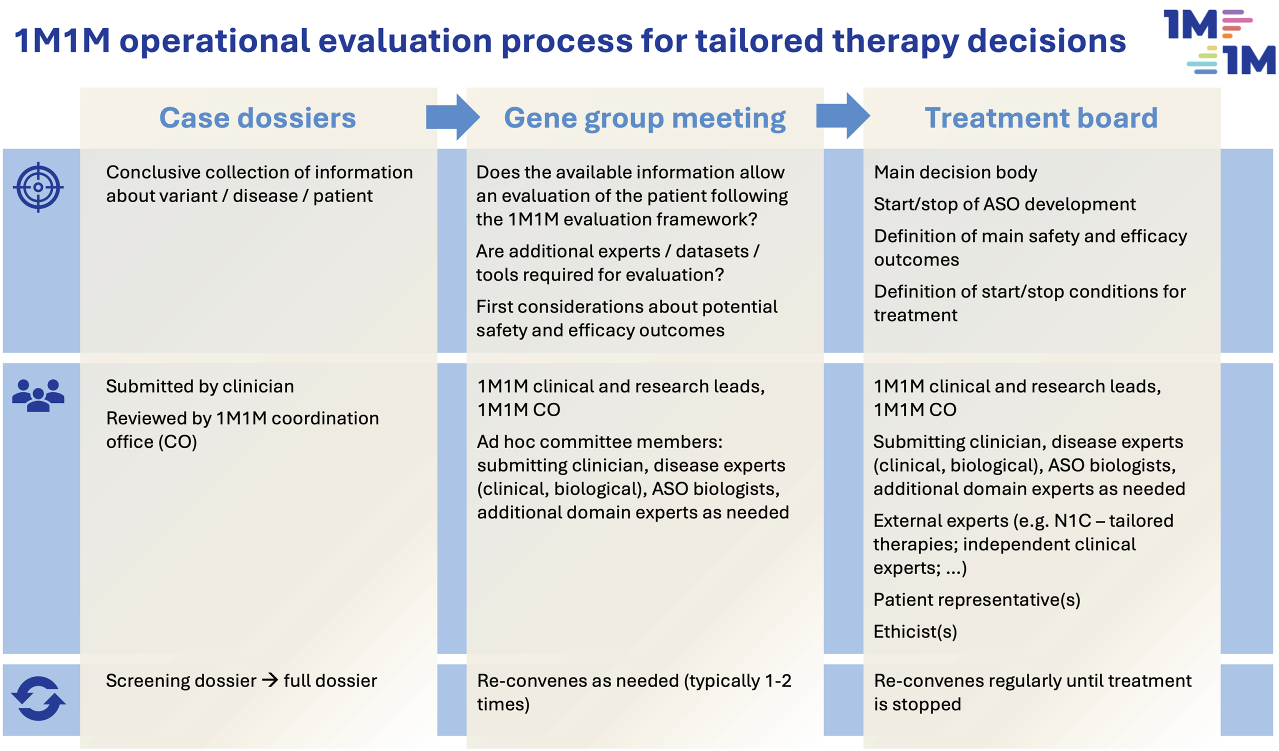

Figure 5. 1M1M operational evaluation process for tailored therapy development
Cases are first evaluated in a two-step process involving a screening dossier and a full dossier (see supplemental information). The gene group then reviews and synthesizes the available information to support decision-making by the multi-stakeholder treatment board.

treatment *in principle* and provide a fact-based counseling perspective to the patient. The treatment board hereby assumes the role of a highly specialized consultation and decision board, complementing local ethics committees or institutional review boards by providing the necessary multi-disciplinary genetic, biological, clinical and patient perspective and in-depth subject matter expertise on the many aspect of the tailored drug development process. Yet, it is ultimately the patient's or family's personal conception of benefit and harm that will determine the final treatment decision. The appropriate timing for involving the patient or family, as well as the individually appropriate manner of information and consenting, are highly complex and, in our experience, require a step-by-step interactive approach. A consenting framework for mutation-specific treatments is warranted but goes beyond the scope of this manuscript.

CONCLUSIONS

The complexity and impact of the decision to develop and apply a tailored RNA therapy warrants a standardized decision approach taking into account multiple perspectives and experiences. The "1 Mutation 1 Medicine" (1M1M; https://www.1mutation1medicine.eu/) consortium has therefore put in place a stringent quality-controlled process comprising three main steps (Figure 5). Figure 6 summarizes real-world experience derived from the evaluation of 59 cases that have been submitted by physicians to the 1M1M network since the best-practice framework was established.

(1) A patient dossier (screening dossier/full dossier; see supplemental information) allows standardized capture of all relevant information on all 3 criteria axes and rapid screening of basic eligibility criteria. (2) Then, a multi-disciplinary gene group is convened which surveys the available information, confirms general eligibility, defines informational gaps, and may draw in additional resources required to make a treatment decision such as additional datasets (e.g., to determine natural history), tools or expertise. (3) Finally, a treatment board is constituted, which consists of the abovementioned multistakeholder team supplemented by patient representatives, ethicists and additional domain experts considered necessary by the gene group. The treatment board is the main decision body, governing not only the initial decision to treat but also the course and potentially termination of treatment. It makes the final decision whether treatment development should commence and treatment should be offered to the patient. Additionally, it defines starting and stopping rules for the treatment based on predetermined efficacy and safety outcomes, thus providing the local treatment team with essential guidance.

Overall, our 1M1M best-practice framework and its associated processes may serve both as blueprint for the systematic evaluation of

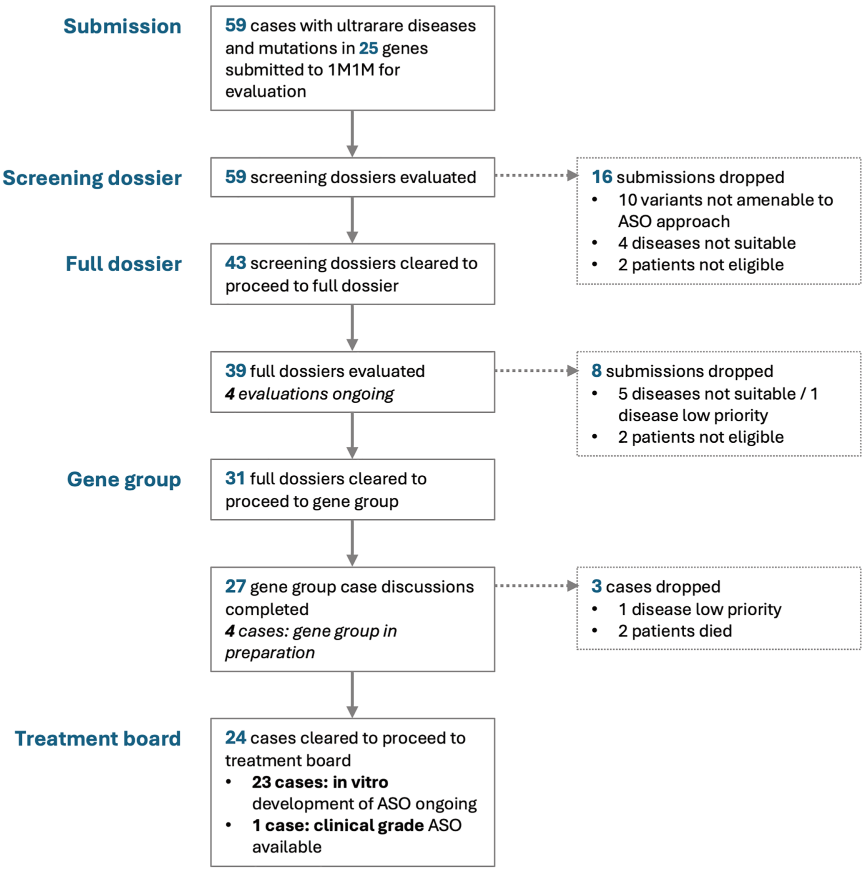

Figure 6. Usage statistics of the 1M1M best practice framework

Since the completion of the framework and implementation of formalized registration of all submitted cases, the framework was applied to the evaluation of 59 cases with ultrarare diseases. The flowchart provides an overview of the number of cases assessed at each stage and the most common reasons for discontinuation of drug development.

patients most suitable for individualized drug development; and as a vision for a future in which the development and application of individualized genomic medicines for rare diseases and mutations becomes routine in academic medical centers.

In this regard, our framework may serve as the nucleus for a future standard-of-care pathway in which individualization becomes routine, and validated processes for individualized drug development complement the validation of a fixed end product.

ACKNOWLEDGMENTS

The authors are grateful to the N = 1 Collaborative and especially the N1C patient selection working group for the extremely fruitful discussions on the topic of RNA therapy development for ultrarare diseases. We also gratefully acknowledge the support of Winston Yan, who reviewed the manuscript as a native English speaker to improve its grammar and style and who furthermore provided valuable substantive suggestions. This work was supported by the European Union via the project European Rare Disease Research Alliance (ERDERA), GA no. 101156595, funded under call HORIZON-HLTH-2023-DISEASE-07 (to R.S., H.G., A.A.-R., and M.S.), as well as via the Horizon Europe grant "Medicine Made to Measures" (MMM; grant 101120256 to R.S., H.G., A.A.-R., W. M.C.v.R.-M., and M.S.). Moreover, this work received financial support from the Department of Human Genetics of the LUMC (W.M.C.v.R.-M. and A.A.-R.); the Federal Ministry of Education and Research, Germany, through funding for the TreatHSP network (01GM2209A to R.S.); and the Clinician Scientist program "PRECISE.net" funded by the Else Kröner-Fresenius-Stiftung (to R.S. and M.S.). Several of the authors are members of the European Reference Network for Rare Neurological Diseases ERN-RND (project ID no. 739510; R.S., H.G., and M.S.).

AUTHOR CONTRIBUTIONS

All authors contributed to the conception and design of the study; R.S. and M.S. drafted the manuscript; all authors critically reviewed the manuscript for important intellectual content.

DECLARATION OF INTERESTS

R.S. is a member of the scientific advisory board of the Genesis Project Foundation, the HSP Research Foundation, and the Our Moon's Mission Foundation.

H.G. has received consultancy honoraria from UCB, unrelated to the present manuscript. H.G. is a member of the think tank of the Eva Luise und Horst Köhler foundation.

For full transparency, A.A.-R. discloses employment by LUMC, which has patents on exon skipping technology for Duchenne muscular dystrophy and other rare diseases, some of which have been licensed to BioMarin and subsequently sublicensed to Sarepta. As a co-inventor of some of these patents, A.A.-R. was entitled to a share of royalties. A.A.-R. further discloses being *ad hoc* consultant for PTC Therapeutics, Sarepta Therapeutics, Regenxbio, Dyne Therapeutics, Lilly, BioMarin Pharmaceutical Inc., Eisai, Entrada, Takeda, Splicesense, Galapagos, and Astra Zeneca. Past *ad hoc* consulting has occurred for Alpha Anomeric, CRISPR Therapeutics, Summit PLC, Audentes, Santhera, BridgeBio, Global Guidepoint and GLG consultancy, Grunenthal, Wave, and BioClinica. A.A.-R. also reports having been a member of the Duchenne Network Steering Committee (BioMarin) and being a member of the scientific advisory boards of Eisai, Hybridize Therapeutics, Silence Therapeutics, and Sarepta therapeutics. Remuneration for these consulting and advisory activities is paid to LUMC. LUMC also received speaker honoraria from PTC Therapeutics, Alnylam Netherlands, Pfizer, and BioMarin Pharmaceutical and funding for contract research from Italfarmaco, Sapreme, Eisai, Galapagos, Synaffix, and Alpha Anomeric. Project funding is received from Sarepta Therapeutics and Entrada.

M.S. has received consultancy honoraria from Ionis, UCB, Prevail, Orphazyme, Servier, Reata, GenOrph, AviadoBio, Biohaven, Zevra, and Lilly, all unrelated to the present manuscript.

SUPPLEMENTAL INFORMATION

Supplemental information can be found online at https://doi.org/10.1016/j.omtn.2025.102615.

REFERENCES

1. Morschhauser, F., Dahiya, S., Palomba, M.L., Martin Garcia-Sancho, A., Reguera Ortega, J.L., Kuruvilla, J., Jäger, U., Cartron, G., Izutsu, K., Dreyling, M., et al. (2024). Lisocabtagene maraleucel in follicular lymphoma: the phase 2 TRANSCEND FL study. Nat. Med. *30*, 2199–2207.
2. European Medicines Agency (2023). Lisocabtagene maraleucel - European Commission decision. https://www.ema.europa.eu/en/medicines/human/EPAR/breyanzi#authorisation-details.
3. U.S. Food & Drug Administration (2024). Lisocabtagene maraleucel - FDA Approved Cellular and Gene Therapy Products. https://www.fda.gov/vaccines-blood-biologics/cellular-gene-therapy-products/breyanzi-lisocabtagene-maraleucel.
4. Fumagalli, F., Calbi, V., Natali Sora, M.G., Sessa, M., Baldoli, C., Rancoita, P.M.V., Ciotti, F., Sarzana, M., Fraschini, M., Zambon, A.A., et al. (2022). Lentiviral haematopoietic stem-cell gene therapy for early-onset metachromatic leukodystrophy: long-term results from a non-randomised, open-label, phase 1/2 trial and expanded access. Lancet *399*, 372–383.
5. European Medicines Agency (2022). Lisocabtagene maraleucel - European Commission decision. https://www.ema.europa.eu/en/medicines/human/EPAR/breyanzi.
6. U.S. Food & Drug Administration (2024). Atidarsagene autotemcel - FDA Approved Cellular and Gene Therapy Products. https://www.fda.gov/vaccines-blood-biologics/cellular-gene-therapy-products/lenmeldy.
7. Adams, D., Gonzalez-Duarte, A., O'Riordan, W.D., Yang, C.C., Ueda, M., Kristen, A. V., Tournev, I., Schmidt, H.H., Coelho, T., Berk, J.L., et al. (2018). Patisiran, an RNAi Therapeutic, for Hereditary Transthyretin Amyloidosis. N. Engl. J. Med. *379*, 11–21.
8. Benson, M.D., Waddington-Cruz, M., Berk, J.L., Polydefkis, M., Dyck, P.J., Wang, A. K., Planté-Bordeneuve, V., Barroso, F.A., Merlini, G., Obici, L., et al. (2018). Inotersen Treatment for Patients with Hereditary Transthyretin Amyloidosis. N. Engl. J. Med. *379*, 22–31.
9. European Medicines Agency (2018). Inotersen - European Commission decision. https://www.ema.europa.eu/en/medicines/human/EPAR/tegsedi.
10. European Medicines Agency (2018). Patisiran - European Commission decision. https://www.ema.europa.eu/en/medicines/human/EPAR/onpattro.
11. Maurer, M.S., Kale, P., Fontana, M., Berk, J.L., Grogan, M., Gustafsson, F., Hung, R. R., Gottlieb, R.L., Damy, T., González-Duarte, A., et al. (2023). Patisiran Treatment in Patients with Transthyretin Cardiac Amyloidosis. N. Engl. J. Med. *389*, 1553–1565.
12. U.S. Food & Drug Administration (2018). Inotersen - Drugs@FDA. https://www.accessdata.fda.gov/scripts/cder/daf/index.cfm?event=BasicSearch.process.
13. U.S. Food & Drug Administration (2018). Patisiran - Drugs@FDA. https://www.accessdata.fda.gov/scripts/cder/daf/index.cfm?event=BasicSearch.process.
14. European Medicines Agency (2024). Tofersen - European Commission decision. https://www.ema.europa.eu/en/medicines/human/EPAR/qalsody.
15. Miller, T.M., Cudkowicz, M.E., Genge, A., Shaw, P.J., Sobue, G., Bucelli, R.C., Chiò, A., Van Damme, P., Ludolph, A.C., Glass, J.D., et al. (2022). Trial of Antisense Oligonucleotide Tofersen for SOD1 ALS. N. Engl. J. Med. *387*, 1099–1110.
16. U.S. Food & Drug Administration (2023). Tofersen - Drugs@FDA. https://www.accessdata.fda.gov/scripts/cder/daf/index.cfm?event=BasicSearch.process.
17. European Medicines Agency (2017). Nusinersen - European Commission decision. https://www.ema.europa.eu/en/medicines/human/EPAR/spinraza.
18. Finkel, R.S., Chiriboga, C.A., Vajsar, J., Day, J.W., Montes, J., De Vivo, D.C., Yamashita, M., Rigo, F., Hung, G., Schneider, E., et al. (2016). Treatment of infantile-onset spinal muscular atrophy with nusinersen: a phase 2, open-label, dose-escalation study. Lancet *388*, 3017–3026.
19. Finkel, R.S., Mercuri, E., Darras, B.T., Connolly, A.M., Kuntz, N.L., Kirschner, J., Chiriboga, C.A., Saito, K., Servais, L., Tizzano, E., et al. (2017). Nusinersen versus Sham Control in Infantile-Onset Spinal Muscular Atrophy. N. Engl. J. Med. *377*, 1723–1732.
20. Mercuri, E., Darras, B.T., Chiriboga, C.A., Day, J.W., Campbell, C., Connolly, A.M., Iannaccone, S.T., Kirschner, J., Kuntz, N.L., Saito, K., et al. (2018). Nusinersen versus Sham Control in Later-Onset Spinal Muscular Atrophy. N. Engl. J. Med. *378*, 625–635.
21. U.S. Food & Drug Administration (2016). Nusinersen - Drugs@FDA. https://www.accessdata.fda.gov/scripts/cder/daf/index.cfm?event=BasicSearch.process.
22. Nguengang Wakap, S., Lambert, D.M., Olry, A., Rodwell, C., Gueydan, C., Lanneau, V., Murphy, D., Le Cam, Y., and Rath, A. (2020). Estimating cumulative point prevalence of rare diseases: analysis of the Orphanet database. Eur. J. Hum. Genet. *28*, 165–173.
23. Beaverson, K.L., Julkowska, D., Letinturier, M.C.V., Aartsma-Rus, A., Austin, J., Bueren, J., Frost, S., Hamamura, M., Larkindale, J., LaRosa, G., et al. (2023). The IRDiRC Chrysalis Task Force: making rare disease research attractive to companies. Ther. Adv. Rare Dis. *4*, 26330040231188979.
24. Aartsma-Rus, A., van Roon-Mom, W., Lauffer, M., Siezen, C., Duijndam, B., Coenen-de Roo, T., Schüle, R., Synofzik, M., and Graessner, H. (2023). Development of tailored splice-switching oligonucleotides for progressive brain disorders in Europe: development, regulation, and implementation considerations. RNA *29*, 446–454.
25. Synofzik, M., van Roon-Mom, W.M.C., Marckmann, G., van Duyvenvoorde, H.A., Graessner, H., Schüle, R., and Aartsma-Rus, A. (2022). Preparing n-of-1 Antisense Oligonucleotide Treatments for Rare Neurological Diseases in Europe: Genetic, Regulatory, and Ethical Perspectives. Nucleic Acid Ther. *32*, 83–94.
26. Center for Drug Evaluation and Research, CfBEaR, Center for Devices and Radiological Health (2023). Patient-Focused Drug Development: Incorporating Clinical Outcome Assessments Into Endpoints for Regulatory Decision-Making. https://www.regulations.gov/docket/FDA-2023-D-0026.
27. Research, CfDEa (2018). Patient-Focused Drug Development: Collecting Comprehensive and Representative Input. https://www.regulations.gov/docket/FDA-2018-D-1893.
28. Research, CfDEa (2022). Patient-Focused Drug Development: Methods to Identify What Is Important to Patients: Guidance for Industry, Food and Drug Administration Staff, and Other Stakeholders. https://www.regulations.gov/docket/FDA-2019-D-4247.
29. Research, CfDEa (2022). Patient-Focused Drug Development: Selecting, Developing, or Modifying Fit-for-Purpose Clinical Outcome Assessments. https://www.regulations.gov/docket/FDA-2022-D-1385.
30. Liu, M., Wang, Y., Zhang, Y., Hu, D., Tang, L., Zhou, B., and Yang, L. (2025). Landscape of small nucleic acid therapeutics: moving from the bench to the clinic as next-generation medicines. Signal Transduct. Target. Ther. *10*, 73.
31. Bereczki, Z., Benczik, B., Balogh, O.M., Marton, S., Puhl, E., Pétervári, M., Váczy-Földi, M., Papp, Z.T., Makkos, A., Glass, K., et al. (2025). Mitigating off-target effects of small RNAs: conventional approaches, network theory and artificial intelligence. Br. J. Pharmacol. *182*, 340–379.
32. Sang, A., Zhuo, S., Bochanis, A., Manautou, J.E., Bahal, R., Zhong, X.B., and Rasmussen, T.P. (2024). Mechanisms of Action of the US Food and Drug Administration-Approved Antisense Oligonucleotide Drugs. BioDrugs *38*, 511–526.
33. Miao, Y., Fu, C., Yu, Z., Yu, L., Tang, Y., and Wei, M. (2024). Current status and trends in small nucleic acid drug development: Leading the future. Acta Pharm. Sin. B *14*, 3802–3817.
34. Gao, X., Diep, J.K., Norris, D.A., Yu, R.Z., and Geary, R.S. (2023). Predicting the pharmacokinetics and pharmacodynamics of antisense oligonucleotides: an overview of various approaches and opportunities for PBPK/PD modelling. Expert Opin. Drug Metab. Toxicol. *19*, 979–990.
35. Kim, J., Hu, C., Moufawad El Achkar, C., Black, L.E., Douville, J., Larson, A., Pendergast, M.K., Goldkind, S.F., Lee, E.A., Kuniholm, A., et al. (2019). Patient-Customized Oligonucleotide Therapy for a Rare Genetic Disease. N. Engl. J. Med. *381*, 1644–1652.

36. Centa, J.L., Stratton, M.P., Pratt, M.A., Osterlund Oltmanns, J.R., Wallace, D.G., Miller, S.A., Weimer, J.M., and Hastings, M.L. (2023). Protracted CLN3 Batten disease in mice that genetically model an exon-skipping therapeutic approach. Mol. Ther. Nucleic Acids *33*, 15–27.

37. Kim, J., Woo, S., de Gusmao, C.M., Zhao, B., Chin, D.H., DiDonato, R.L., Nguyen, M.A., Nakayama, T., Hu, C.A., Soucy, A., et al. (2023). A framework for individualized splice-switching oligonucleotide therapy. Nature *619*, 828–836.

38. Crooke, S.T., Cole, T., Carroll, J., Gleeson, J.G., Mignon, L., Douville, J., Chung, W., Bain, J., Berry-Kravis, E., Leung, N., et al. (2024). Genotypic and phenotypic analysis of 173 patients with extremely rare pathogenic mutations who applied for experimental antisense oligonucleotide treatment. Preprint at: medRxiv. https://doi.org/10.1101/2024.08.05.24310862

39. Wagner, M., Berecki, G., Fazeli, W., Nussbaum, C., Flemmer, A.W., Frizzo, S., Heer, F., Heinen, F., Horton, R., Jacotin, H., et al. (2025). Antisense oligonucleotide treatment in a preterm infant with early-onset SCN2A developmental and epileptic encephalopathy. Nat. Med. https://doi.org/10.1038/s41591-025-03656-0.

40. Ziegler, A., Carroll, J., Bain, J.M., Sands, T.T., Fee, R.J., Uher, D., Kanner, C.H., Montes, J., Glass, S., Douville, J., et al. (2024). Antisense oligonucleotide therapy in an individual with KIF1A-associated neurological disorder. Nat. Med. *30*, 2782–2786.

41. Wilton-Clark, H., Yan, E., and Yokota, T. (2024). Preparing for Patient-Customized N-of-1 Antisense Oligonucleotide Therapy to Treat Rare Diseases. Genes *15*, 821.

42. Hosaka, T., Tsuji, H., and Kwak, S. (2021). RNA Editing: A New Therapeutic Target in Amyotrophic Lateral Sclerosis and Other Neurological Diseases. Int. J. Mol. Sci. *22*, 10958.

43. McKenzie, H. (2025). New Hope for Rare and Common Diseases. https://www.biospace.com/drug-development/rna-editing-hits-the-clinic-fueling-new-hope-for-rare-and-common-diseases.

44. Farag, M., Tabrizi, S.J., and Wild, E.J. (2025). Huntington's disease clinical trials update: March 2025. J. Huntingtons Dis. *14*, 18796397251337000.

45. Monfrini, E., Baso, G., Ronchi, D., Meneri, M., Gagliardi, D., Quetti, L., Verde, F., Ticozzi, N., Ratti, A., Di Fonzo, A., et al. (2024). Unleashing the potential of mRNA therapeutics for inherited neurological diseases. Brain *147*, 2934–2945.

46. Feng, S., Rcheulishvili, N., Jiang, X., Zhu, P., Pan, X., Wei, M., Wang, P.G., Ji, Y., and Papukashvili, D. (2024). A review on Gaucher disease: therapeutic potential of beta-glucocerebrosidase-targeted mRNA/saRNA approach. Int. J. Biol. Sci. *20*, 2111–2129.

47. Dowling, J.J., Pirovolakis, T., Devakandan, K., Stosic, A., Pidsadny, M., Nigro, E., Sahin, M., Ebrahimi-Fakhari, D., Messahel, S., Varadarajan, G., et al. (2024). AAV gene therapy for hereditary spastic paraplegia type 50: a phase 1 trial in a single patient. Nat. Med. *30*, 1882–1887.

48. Yu, T.W., and Bodamer, O. (2022). A solid start for gene therapy in Tay-Sachs disease. Nat. Med. *28*, 236–237.

49. Brooks, P.J., Miller, T.M., Revah, F., Suh, J., Garrison, B.R., Starke, L.C., MacLachlan, T.K., Neilan, E.G., Raychaudhuri, G., Kassim, S.H., et al. (2024). The Bespoke Gene Therapy Consortium: facilitating development of AAV gene therapies for rare diseases. Nat. Rev. Drug Discov. *23*, 157–158.

50. Health, FftNIo, and Partnership, A.M. (2025). AMP® Bespoke Gene Therapy Consortium (BGTC) Regulatory Playbook (last updated: May 2025) (FNIH).

51. Musunuru, K., Grandinette, S.A., Wang, X., Hudson, T.R., Briseno, K., Berry, A.M., Hacker, J.L., Hsu, A., Silverstein, R.A., Hille, L.T., et al. (2025). Patient-Specific In Vivo Gene Editing to Treat a Rare Genetic Disease. N. Engl. J. Med. *392*, 2235–2243.

52. Lim, C.K.W., Gapinske, M., Brooks, A.K., Woods, W.S., Powell, J.E., Zeballos C, M. A., Winter, J., Perez-Pinera, P., and Gaj, T. (2020). Treatment of a Mouse Model of ALS by In Vivo Base Editing. Mol. Ther. *28*, 1177–1189.

53. Shirguppe, S., Gapinske, M., Swami, D., Gosstola, N., Acharya, P., Miskalis, A., Joulani, D., Szkwarek, M.G., Bhattacharjee, A., Elias, G., et al. (2024). In vivo CRISPR base editing for treatment of Huntington's disease. Preprint at: bioRxiv. https://doi.org/10.1101/2024.07.05.602282

54. Chen, S., Heendeniya, S.N., Le, B.T., Rahimizadeh, K., Rabiee, N., Zahra, Q.U.A., and Veedu, R.N. (2024). Splice-Modulating Antisense Oligonucleotides as Therapeutics for Inherited Metabolic Diseases. BioDrugs *38*, 177–203.

55. Stern, S., Wange, R.L., and Rogers, H. (2024). An Evaluation of First-in-Human Studies for RNA Oligonucleotides. Nucleic Acid Ther. *34*, 276–284.

56. Richards, S., Aziz, N., Bale, S., Bick, D., Das, S., Gastier-Foster, J., Grody, W.W., Hegde, M., Lyon, E., Spector, E., et al. (2015). Standards and guidelines for the interpretation of sequence variants: a joint consensus recommendation of the American College of Medical Genetics and Genomics and the Association for Molecular Pathology. Genet. Med. *17*, 405–424.

57. Lim, K.H., Han, Z., Jeon, H.Y., Kach, J., Jing, E., Weyn-Vanhentenryck, S., Downs, M., Corrionero, A., Oh, R., Scharner, J., et al. (2020). Antisense oligonucleotide modulation of non-productive alternative splicing upregulates gene expression. Nat. Commun. *11*, 3501.

58. Lauffer, M.C., van Roon-Mom, W., and Aartsma-Rus, A.; N = 1 Collaborative (2024). Possibilities and limitations of antisense oligonucleotide therapies for the treatment of monogenic disorders. Commun. Med. *4*, 6.

59. Aartsma-Rus, A., and den Dunnen, J.T. (2019). Phenotype predictions for exon deletions/duplications: A user guide for professionals and clinicians using Becker and Duchenne muscular dystrophy as examples. Hum. Mutat. *40*, 1630–1633.

60. Hoffman, E.P. (2020). Causes of clinical variability in Duchenne and Becker muscular dystrophies and implications for exon skipping therapies. Acta Myol. *39*, 179–186.

61. Tabrizi, S.J., Estevez-Fraga, C., van Roon-Mom, W.M.C., Flower, M.D., Scahill, R.I., Wild, E.J., Muñoz-Sanjuan, I., Sampaio, C., Rosser, A.E., and Leavitt, B.R. (2022). Potential disease-modifying therapies for Huntington's disease: lessons learned and future opportunities. Lancet Neurol. *21*, 645–658.

62. Jafar-Nejad, P., Powers, B., Soriano, A., Zhao, H., Norris, D.A., Matson, J., DeBrosse-Serra, B., Watson, J., Narayanan, P., Chun, S.J., et al. (2021). The atlas of RNase H antisense oligonucleotide distribution and activity in the CNS of rodents and non-human primates following central administration. Nucleic Acids Res. *49*, 657–673.

63. Liu, M., Fields, F.O., Prescott, J.S., Bello, A., Bower, N., Darakjy, S., Hartke, J., Kadambi, V., Lapadula, D., Stoch, A., and Derzi, M. (2020). Evaluation of Therapeutics for Severely Debilitating or Life-Threatening Diseases or Conditions: Defining Scope to Enable Global Guidance Development. Clin. Pharmacol. Ther. *107*, 514–520.

64. Wolf, N.I., van Spaendonk, R.M.L., Hobson, G.M., and Kamholz, J. (1993). PLP1 Disorders. In GeneReviews((R)), M.P. Adam, G.M. Mirzaa, and R.A. Pagon, eds. (University of Washington).

65. Wambach, J.A., Wegner, D.J., Patni, N., Kircher, M., Willing, M.C., Baldridge, D., Xing, C., Agarwal, A.K., Vergano, S.A.S., Patel, C., et al. (2018). Bi-allelic POLR3A Loss-of-Function Variants Cause Autosomal-Recessive Wiedemann-Rautenstrauch Syndrome. Am. J. Hum. Genet. *103*, 968–975.

66. Paolacci, S., Li, Y., Agolini, E., Bellacchio, E., Arboleda-Bustos, C.E., Carrero, D., Bertola, D., Al-Gazali, L., Alders, M., Altmüller, J., et al. (2018). Specific combinations of biallelic POLR3A variants cause Wiedemann-Rautenstrauch syndrome. J. Med. Genet. *55*, 837–846.

67. Minnerop, M., Kurzwelly, D., Wagner, H., Soehn, A.S., Reichbauer, J., Tao, F., Rattay, T.W., Peitz, M., Rehbach, K., Giorgetti, A., et al. (2017). Hypomorphic mutations in POLR3A are a frequent cause of sporadic and recessive spastic ataxia. Brain *140*, 1561–1578.

68. Motyl, A.A.L., and Gillingwater, T.H. (2022). Timing is everything: Clinical evidence supports pre-symptomatic treatment for spinal muscular atrophy. Cell Rep. Med. *3*, 100725.

69. Sperling, R.A., Donohue, M.C., Raman, R., Rafii, M.S., Johnson, K., Masters, C.L., van Dyck, C.H., Iwatsubo, T., Marshall, G.A., Yaari, R., et al. (2023). Trial of Solanezumab in Preclinical Alzheimer's Disease. N. Engl. J. Med. *389*, 1096–1107.

70. Devanarayan, V., Doherty, T., Charil, A., Sachdev, P., Ye, Y., Murali, L.K., Llano, D. A., Zhou, J., Reyderman, L., Hampel, H., et al. (2024). Plasma pTau217 predicts continuous brain amyloid levels in preclinical and early Alzheimer's disease. Alzheimers Dement. *20*, 5617–5628.

71. Benatar, M., Wuu, J., Andersen, P.M., Bucelli, R.C., Andrews, J.A., Otto, M., Farahany, N.A., Harrington, E.A., Chen, W., Mitchell, A.A., et al. (2022). Design

of a Randomized, Placebo-Controlled, Phase 3 Trial of Tofersen Initiated in Clinically Presymptomatic SOD1 Variant Carriers: the ATLAS Study. Neurotherapeutics *19*, 1248–1258.

72. Research, CfDEa (2021). IND Submissions for Individualized Antisense Oligonucleotide Drug Products for Severely Debilitating or Life-Threatening Diseases: Clinical Recommendations (U.S. FDA).
73. Research, CfDEa (2021). IND Submissions for Individualized Antisense Oligonucleotide Drug Products for Severely Debilitating or Life-Threatening Diseases: Chemistry, Manufacturing, and Controls Recommendations (U.S. FDA).
74. Research, CfDEa (2021). Nonclinical Testing of Individualized Antisense Oligonucleotide Drug Products for Severely Debilitating or Life-Threatening Diseases (U.S. FDA).
75. Research, CfDEa (2021). IND Submissions for Individualized Antisense Oligonucleotide Drug Products: Administrative and Procedural Recommendations (U.S. FDA).
76. Bonifert, T., Karle, K.N., Tonagel, F., Batra, M., Wilhelm, C., Theurer, Y., Schoenfeld, C., Kluba, T., Kamenisch, Y., Carelli, V., et al. (2014). Pure and syndromic optic atrophy explained by deep intronic OPA1 mutations and an intralocus modifier. Brain *137*, 2164–2177.
77. Synofzik, M., and Marckmann, G. (2008). Dein Wille geschehe? Die Pluralität evaluativer Vorstellungen eines gelingenden Lebens und die Fürsorgepflichten des Arztes. In Pluralität in der Medizin Werte-Methoden-Theorien, S. Michels, T. Potthast, and U. Wiesing, eds. (Alber), pp. 271–290.

OMTN, Volume *36*

Supplemental information

Tailored antisense oligonucleotides for ultrarare CNS diseases: An experience-based best practice framework for individual patient evaluation

Rebecca Schüle, Holm Graessner, Annemieke Aartsma-Rus, Willeke M.C. van Roon-Mom, N=1 Collaborative (N1C), 1 Mutation 1 Medicine Consortium (1M1M), and Matthis Synofzik

Supplemental Material

1M1M Screening dossier.

The screening dossier is designed to enable a first rapid screening evaluation whether a submitted case is in principle eligible for an individualized ASO therapy approach, considering properties of the variant, disease, and patient. After positive evaluation of the screening dossier, a more detailed case evaluation is performed based on the full dossier.

1M1M Full dossier.

The full dossier is designed to capture all key information that is required to perform an individual benefit-risk evaluation, determining the suitability of the respective individual for an individualized treatment approach. The depth of information goes considerably beyond the information provided in the screening dossier. Drawing on the information provided here, a multi-stakeholder gene group will deliberate on whether the current data is adequate for the evaluation process by a treatment board. If deemed insufficient, the group will identify the specific types of additional information required.
